# Dietary habits and supplementation practices of young women during pregnancy: an online cross-sectional survey of young mothers and health care professionals

**DOI:** 10.1186/s40795-017-0137-3

**Published:** 2017-03-01

**Authors:** Hora Soltani, Alexandra Duxbury, Rachel Rundle, Katie Marvin-Dowle

**Affiliations:** 1 Maternal and Infant Health, Centre for Health and Social Care Research, Sheffield Hallam University, Sheffield, UK; 2 Public Health Nutrition, Food and Nutrition Group, Sheffield Business School, Sheffield Hallam University, Sheffield, UK; 3 Centre for Health and Social Care Research, Sheffield Hallam University, Sheffield, UK

**Keywords:** Nutrition, Supplements, Adolescent pregnancy, Midwives, Healthy eating information sources, Young mother, Digital

## Abstract

**Background:**

Nutrition is a modifiable factor affecting birth outcomes, particularly in adolescent pregnancies. This study explores diet and supplementation practices, information and advice before, during and after pregnancy from the perspectives of pregnant or new young mothers and healthcare professionals.

**Methods:**

Two cross-sectional surveys used online questionnaires for young women who were currently pregnant or who had recently given birth, and health care professionals providing antenatal care. The surveys utilised a combination of question types including free text and multiple choice. Recruitment was conducted via the Tommy's website, online forums for young mothers and professional networks.

**Results:**

A total of 205 young women and 146 health care professionals were included in the study.

Most young women reported taking supplements at some stage of pregnancy (93.2%), with 54.6% taking it on a daily basis. Those who reported taking supplements less than 7 days a week stated it was mainly due to forgetting. Health care professionals however reported that some young women had difficulties accessing healthy start supplements. Young women reported positive dietary changes; however a significant proportion of participants indicated that they avoided some foods unnecessarily. Avoiding or reducing foods such as red meat (22.7%), eggs (40.6%), oily fish (60.4%) and soft cheese (36.2%) is of concern. Midwife/family nurse (38.0%) was young women's current favourite information source; smartphone applications (apps) and recipe booklets were suggested by over 50% of participants as a new addition to existing services. Health care professionals reported they included nutritional information and support as part of their role; however they felt there were some gaps in knowledge and confidence. Midwives in particular suggested a lack of sufficient time and resources as a main challenge in providing adequate support.

**Conclusions:**

Avoiding or reducing major food groups was reported which can indicate a poor dietary pattern. A positive change in dietary intake reported by the majority of young women in this survey indicates willingness to adopt a healthy lifestyle. This, in addition to their trust in health professionals particularly midwives, provides an opportunity for health interventions which support behaviour change to improve birth outcomes. Identified gaps in knowledge and confidence by health professionals in providing dietary advice highlight the need for some additional training for health professionals in delivering dietary and lifestyle behaviour change interventions. Independent and trustworthy online resources for women and their health professionals which can be accessed at any time to provide up to date information in between appointments are also required.

## Background

A healthy diet and lifestyle during pregnancy is essential to ensure fetal growth and development is optimised. This is of a particular importance for young adolescent mothers in whom poor nutrition [1] and adverse outcomes such as stillbirth, preterm birth and low birthweight have been frequently reported [2–4]. Adolescent pregnancy (birth among women aged 15–19 when the pregnancy ended) has been considered a major contributor to maternal and child mortality worldwide [5] and is a major public health concern in United Kingdom (UK) with a rate of 19.7 births per 1000 women [6].

Young or adolescent mothers have specific requirements for macro- and micronutrients to support fetal development in addition to their own growing needs [7]. Systematic evidence [1] in addition to the recent UK ‘National Diet and Nutrition Survey’ (NDNS) [8] highlight that many adolescents' diets do not provide the recommended intakes for iron, zinc, calcium and folate and that those with the lowest incomes had significantly lower mean intakes than those from the highest income groups. Spending money on healthy food is often a lower priority compared to other fixed financial pressures such as housing, transport and utility bills [9]. With many competing priorities, it can be a challenge for health professionals to support women in making positive dietary changes and providing practical advice such as guiding on recipes that are suitable for circumstances with limited budget, cooking skills or facilities.

The Department of Health guidance and National Health Service (NHS) Choices [10] website provide standard healthy eating and food safety information for pregnant women in the UK. However most of the routinely provided information is not tailored to the specific nutritional requirements of adolescent mothers or in an adolescent friendly format. Midwives traditionally give every pregnant woman ‘The NHS Pregnancy Guide’, but the 200 page printed book can now only be read online so is likely to be only read by the most motivated of mothers with internet access. Tommy’s, a UK baby charity, has produced the ‘Young Woman’s Guide to Pregnancy’ which provides dietary advice in a booklet format more accessible to adolescents, but distribution of this free resource by midwives is variable [11]. It is therefore paramount to explore where and how young women access information and their preferences so that future resources are more appropriate to their needs.

Young mothers in the UK are entitled to standard antenatal care, although they are more likely to present for their first appointment later and miss appointments [12]. Various models of care are provided for adolescent pregnant mothers. In some areas, specialist teenage pregnancy midwives provide all or some of their care which may involve slightly longer appointment times and signposting to other services such as parenting classes. The Family Nurse Partnership (FNP) scheme provides intensive support for the young pregnant mother from 14 weeks and family for up to 2 years postnatal but only the most vulnerable are eligible and it is not available nationally. Understanding how young women are supported and guided regarding nutrition and healthy eating during pregnancy, and how they would like to be supported in future is essential to optimise care and enhance birth outcomes for this vulnerable group of women.

A previous study surveying adolescent's views of healthy eating during pregnancy in the USA [13] found that while young women reported that they considered healthy eating to be important the majority of their snacks consisted of unhealthy foods. The results from this study are however unclear in terms of the foods eaten at meal times and the frequency of consuming fruit and vegetables. There are no previous studies of this type undertaken in the UK, nor have any studies been conducted which include exploring the views of health professionals' dietary advice and support for this particular vulnerable group of women.

In an in-depth qualitative study [11] we have explored dietary patterns and supplementation adherence, and the facilitators and barriers in achieving a healthy diet and lifestyle during adolescent pregnancy. This qualitative study suggested that many young mothers have a dietary pattern consisting of high fat snacks, carbonated drinks and occasional meals, but their diet often depends on their living arrangements with those still at home more likely to have more traditional eating habits. A larger study was required to verify these provisional findings with a specific focus on what, if any, changes young women made to their diets during pregnancy; what motivated them to make such changes; where they obtained information and how in future they would like to receive information and support regarding diet and supplements during pregnancy. This study therefore aimed to explore diet and supplementation practices, information and advice during pregnancy from the perspectives of adolescent pregnant or new mothers and their health care professionals.

## Methods

Two cross-sectional surveys were developed, one using a questionnaire which was specifically designed, piloted and administered for young women and the other for health care professionals. The questionnaires were informed by the findings from our previous qualitative study on exploring views of adolescent pregnant women and their health care professionals on pregnant adolescents' dietary pattern and, facilitators and barriers for improvement [11].

Ethical approval was obtained from Sheffield Hallam University Research Ethics Committee. An information sheet was provided at the beginning of the questionnaires, providing information about the study, contact details for further information. Young women were invited to participate if they were aged between 16–20 years of age at the time of their pregnancy, with a recent pregnancy experience. Health care professionals were invited to participate if their role included providing antenatal care to adolescent pregnant women. Consent was assumed inherent for the respondents who completed the questionnaire voluntarily.

Survey design: Questions were drafted and formatted in SurveyMonkey and results downloaded for analysis into SPSS (Version 23). The young women's questionnaire was designed to gather data from young women who were currently pregnant or those who had been pregnant recently, so care was taken to word the questions and question logic in order to capture all possibilities. There were a total of 26 questions in the young women’s survey and 25 in the survey for health care professionals both using a mixture of question types, including some free text questions and some where participants selected one from a list of options and matrices. Demographic data was collected from the young women including the first part of their postcode, age and number of pregnancies and, job role, grade and year of qualification were collected from health care professionals.

Questions for young women focussed around changes to their diet and supplement use, beliefs about why they made changes, current information sources and suggestions for future resources or services. Questions for health care professionals focused on when and how information and advice on diet, nutrition and supplements was offered to young women and their confidence in giving this advice.

### Piloting

The surveys were developed in consultation with the young women at the local Young Men Christian Association (YMCA: a global organisation aiming to put principals into practice in order to promote healthy body, mind and spirit) training centre and health professionals at their place of work. The questionnaire was also sent to the project steering group including experts in the field whose comments and suggestions were used to further refine the survey. The survey was updated based on the feedback received such as providing examples where terms identified as complex (e.g. for the term red meat we provided examples for clarity) and reduced the length of time required for questionnaire completion to 10–15 min.

#### Sampling/Recruitment

Tommy’s (the UK based baby charity) facilitated dissemination of the online survey through its well-established networking systems with a wide range of organisations and support groups for young women and their professionals in the UK. The survey was distributed by email and website links on the 23rd of November 2013 from which participants were invited to the study. Implicit consent was presumed by participants following a link and completing the questionnaire.

## Data analysis

Descriptive statistics are reported for all demographic data and for closed answer questions including proportions, means, standard deviation (SD) and ranges as appropriate. Average score or proportions in percent are presented for individual survey items. A simple content analysis was used for the open ended questions to establish categories.

## Results

Young Women’s Questionnaire.

## Respondent characteristics

The sample consisted of 212 young women who started the survey with 162 completing it (76%), most within 48 h of it being posted online. Of 212 original participants, five were excluded as they indicated they have never been pregnant and two because they were under 16 years of age. Characteristics of participants in the survey are presented in Table 1. About 74% of the respondents were over 20 years and 26% aged 16–20 years.

**Table 1:** Characteristics of young women participating in the study (*N* = 205)

	Number	Percent
Age		
16–19 years	23	11.2
20 years	31	15.1
Over 20 years	151	73.7
Ethnicity		
White	194	94.6
Black	2	1.0
Asian	4	2.0
Chinese/other	0	0.0
Mixed	2	1.0
Not stated	3	1.5
Gravida		
Gravida 1	124	60.5
Gravida 2	50	24.4
Gravida 3 or more	29	14.1
Missing	2	1.0
Living arrangements		
Living with partner	131	63.9
Living with my family	36	17.6
Living with my partner's family	8	3.9
Living alone	26	12.7
Living in shared accommodation/hostel	2	1.0
Missing	2	1.0

The majority of respondents (94.6%) described their ethnicity as White British and a small proportion identified themselves as Black, Asian or Mixed race (Table 1).

About 60% of participants had only one pregnancy and the remaining had two or more pregnancies. Fifty-eight women were currently pregnant, ranging from 3 to 40 weeks (mean 25.6, SD10.2) with 30 as their first pregnancy.

Valid postcode district data was available for 180 participants, of whom 141 were resident in England, 20 in Scotland, 15 in Wales and 4 in Northern Ireland [14].

## Use of supplements

**Fig. 1:**
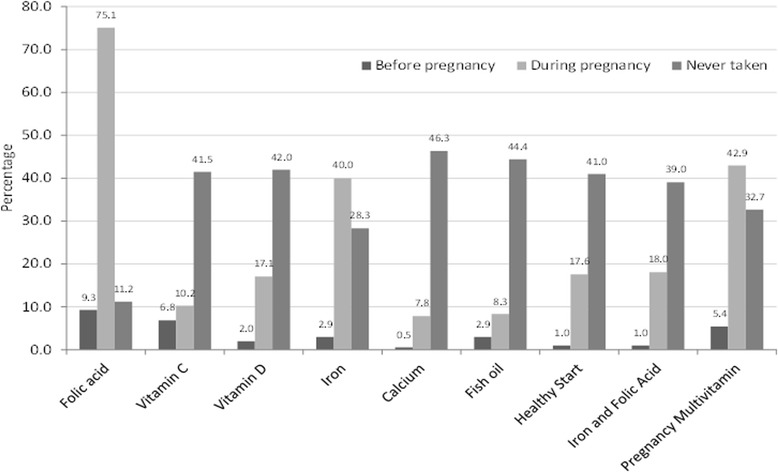
Supplement intake before and during pregnancy by adolescent mothers (*N* = 205)

Young women were asked if they had taken any supplements before, during and/or after their pregnancy, with 93.2% reporting taking supplements at any stage of pregnancy and 5.8% stated they have never taken supplements during pregnancy. Folic acid was the most commonly used supplement with 75.1% stated having taken it at some stage in their pregnancy, followed by pregnancy multivitamins (42.9%) and iron supplements (40.0%) also being prevalent (Fig. 1).

A total of 84.5% took a folic acid supplement before or during their pregnancy, although the remainder might be getting their folic acid from Healthy Start (18.6%), a combined form of iron and folic acid (19.0%) or pregnancy multivitamins (48.3%) in this period.

Out of 205 participants, 54.6% stated that on average they took supplements daily, 24.9% took it 5–6 days a week, 12.2% 1–4 days a week, 4.4% never and 1% didn't know. Of 87 (out of 205) who reported taking supplements less than 7 days a week, 88.5% said it was mainly due to 'forgetting' and only a few stated it to be because of other reasons such as making them feel unwell, being too expensive, not liking to take tablets or not knowing what they are for.

## Food choices and dietary changes

**Fig. 2:**
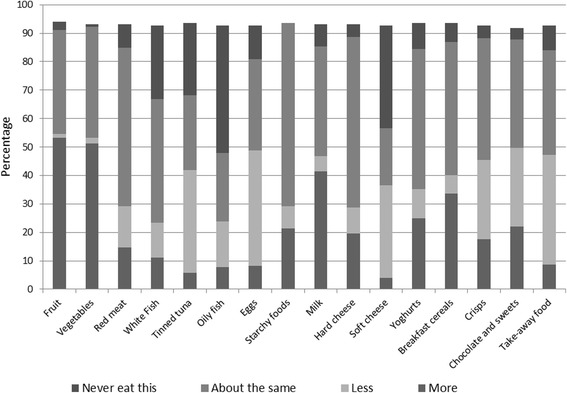
Pattern of dietary changes during pregnancy (*N* = 205)

When young women were asked if they made changes to their diet during pregnancy 78.0% indicated yes, 17.6% said no, 0.5% (1 participant) said don’t know and the remaining (3.9%) did not answer this question. As presented in Fig. 2, the respondents reported consumption of more fruit (53.2%), vegetables (51.2%), milk (41.5%) and breakfast cereal (33.7%). However a considerable proportion of participants indicated that they ate less or never ate red meat (22.9%), eggs (52.2%), oily fish (61.0%) and soft cheese (68.8%).

Table 2 shows that for many young mothers, the main contributing factor for changing to a healthier diet and supplement intake during pregnancy is the connection between healthy diet, supplementation and its impact on foetal growth and development.

**Table 2:** Participants who agree to each statement regarding their reasons for eating a healthy diet or supplement use during pregnancy (*N* = 205)

	Eating well during pregnancy will…	Taking supplements during pregnancy will…
Help me to gain less weight	51.7%	3.9%
Help my baby grow properly	77.6%	71.7%
Help my baby to be healthier after it is born	76.1%	69.8%
Help my baby be more intelligent	20.5%	22.9%
Please my midwife/family nurse	51.2%	59.5%
Please my partner/family	39.0%	45.4%
Cost a fortune	25.9%	31.7%
Be hard work	23.4%	14.6%
Prepare my body for breastfeeding	62.4%	56.1%

## Sources of information and support

**Fig. 3:**
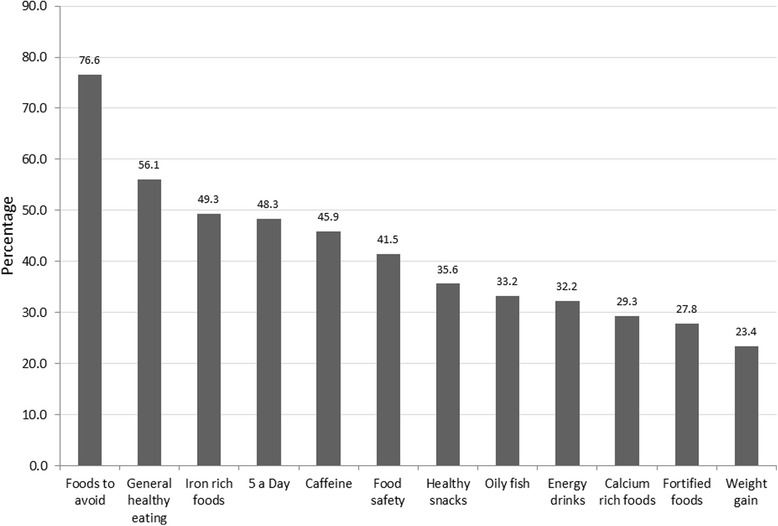
Types of nutrition related information received by young pregnant women during pregnancy (*N* = 205)

As presented in Fig. 3, a great majority of young women remembered receiving information on food to avoid (76.6%) but only around a third or less of them stated that they received information on increasing calcium intake, safe intake of oily fish or weight gain. When they were asked about the main current sources of healthy eating information, they identified midwives (68%), Bounty pack (50%), family (39%) and websites (e.g. Baby Centre, YouTube) (37%) which reflected the state of practice.

**Fig. 4:**
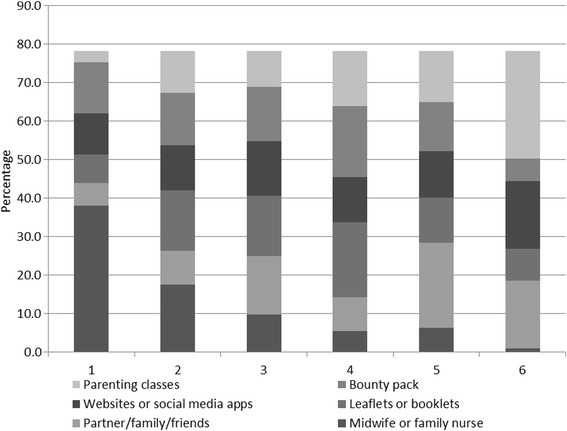
Where young women would like to receive information during pregnancy* (*N* = 205). *: Participants were asked to put the above 6 options in order of preference, with 1 as the most and 6 the least favourite options

When asked to rank their favourite sources of information from a list of 6 (Fig. 4), midwives and family nurse practitioners were the most popular with 38.0% selecting it as their favourite and parenting classes were the least popular (2.9%).

**Fig. 5:**
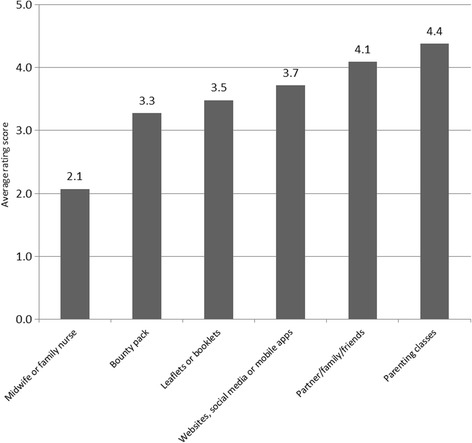
Average scores based on young women’s preferences of information sources during pregnancy* (*N* = 205). *: Participants were asked to put the above 6 options in order of preference, with 1 as the most and 6 the least favourite options

The average rating scores are also calculated from the proportion of respondents who ordered the 6 information source options based on their preferences (Fig. 5), confirming midwives/FNPs as the highly popular source of information (2.1), theoretical sources of information (Bounty pack, leaflets and websites) in the middle with an average score around 3, followed by partner/family and antenatal classes (scored around 4) to be the least favourite options for this group of women.

The free text responses about participants’ views on favourite information sources are summarised in Table 3 under major categories relating to two major themes of advantages and disadvantages. The main advantages of receiving information from health care professionals were indicated to be related to its trustworthiness, reliability, being face to face and that it is through building relationship. The respondents however, highlighted issues such as lack of sufficient information or time as disadvantages for health care professionals to be able to communicate information effectively. There were also those who liked books or websites where they could access information in their own time or Bounty pack free samples. Some had concerns about the accuracy of information on websites or lack of a universal access to internet. Least popular information sources included parenting classes which some respondents thought were patronising, hard to access or felt judged.

**Table 3:** Young women’s views about sources of information in pregnancy

Sources of information	Advantages	Disadvantages
Health care professionals: Midwife or Family Nurse Partnership	• Trust in Professionals *"My midwife has been doing her job for a long time and I trust her, her knowledge and her judgement"* *"Midwives are qualified and know exactly what they're talking about."* • Accurate and reliable advice *"Midwife know everything what is needed for a healthy pregnancy, she was easy to talk to and very informative"* *"Midwife/Family nurse will have knowledge from training, be more understanding of personal circumstance."* • Face to Face information/questions *"Face to face. Factual and can ask questions and get answers immediately!"* *"The midwife discusses it in more detail."* • Continuous relationship through pregnancy *"My first two pregnancies I had the same midwife, I developed a bond with her in this time and felt I could trust the information that has been given to me."* *"More personal; through experience; they know you better"*	• Not getting sufficient information *"I want information off the midwifes but they haven't given me any"* • Appointment length short *"Pre booked appointment to talk more than seven minutes".*
Self- Study: Leaflet/Booklet/Tommy's Guide	• Easy to read in own time *"I can read through in my own time"* *"Can keep for reference"*	• Lose leaflets *"It's easy to lose bits of paper like booklets and guides"* *"They are easy to misplace and lose"*
Bounty pack	• Good timing and free goodies *"They are given to you at an early stage"* *"Creative and lots of goodies with it"* • Universal *"Everyone gets a bounty pack so everyone gets same info."*	• Too much information/reading *"I would not read a whole book on how to do something"* • Advertising & Commercial (Bounty) *Bounty is a marketing scheme so sometimes sceptical on information as they may be trying to sell things to you*
Websites/Social media/Apps	• Accessible anytime *"I can gain the information straight away on my phone instead of having to wait to see your midwife to hand you tonnes of stupid pointless leaflets!"* *"Its easy to get hold of the info 24/7"* "*It's easy access, for instance if I'm not sure wether a certain food is safe to eat during pregnancy I try to look online for the answer."* *"As my social media sites are always on so I can easily access it wherever I am"* *"You can access it anytime without feeling you are constantly hassling someone"*	• Unreliable and contradictory information *"Would have liked more information from midwife as you feel it's trusted information coming from a healthcare provider, whereas on the internet you can receive conflicting information"* *"May not be a reliable source"* *"Cannot always trust the Internet as some sites give opposite advice"* *"Some websites can contradict what you have read on other websites"* • Not accessible to all *"Not everybody has access to them"* *"My phone and tablet don't work very well so I was unable to use apps"*
Social support: Partner/Family/Friends	• Available from day 1 *Family cos you change your habits of eating unhealthy the day you find out your pregnant* • Having experience and knowing you *"family told me information because they have loads of experi with preganices and what to do before getting pregnant with vitamins and eating and drinking after your body and baby"*	• Interfering and patronising *"I couldn't have family advising me, I would find it patronizing."* *"Gets frustrating with family interfering"* *"Don't like being told what to do"* • Outdated/inaccurate advice *"What family friends and partners tell you is not always true and is mainly their opinion"* *"They all have different views",* *"A lot of advice you get giving my family, friends etc. are normally 'old wives tales' that are untrue."*
Parenting Classes	• Social way to meet other mums *Can all talk about different healthy foods in the classes*	• Content too general *"Never told us about healthy eating focus was on labour and delivery"* • Fear/experience of being judged by other mums and session leaders *"Went once felt inadequate, woman was too patronizing and didn't return"* *"Other mums and leader judging different lifestyles, wouldnt have the confidence to speak in one of those classes"* • Time to attend *"I didn't have time to attend parenting classes when pregnant"* *"Not everyone goes to classes and theres alot to take in"* *"Dislike the parenting class atmosphere."*

Finally, young women were asked to choose three options from a list of 9 suggestions for developing a new supporting tool to provide information about eating a healthy diet and supplements during pregnancy. These suggestions came from the earlier qualitative study and were included for verification in this study with a larger sample. Table 4, shows that the apps (52.7%) and recipe booklets (51.2%) were the most popular options for future developments.

**Table 4:** Responses to the question *“If we could develop something new to provide information about eating a healthy diet and supplements to support young pregnant women, what do you think would be best”* (*N* = 205)

	Young women’s choices
App containing healthy eating information	52.7%
Recipe booklet showing healthy meals and snacks to be eaten during pregnancy	51.2%
Education session to be delivered at antenatal/parenting classes	31.2%
Young woman specific booklet	30.2%
Education session to be delivered in secondary schools/colleges	22.4%
Wallet sized fact sheet/reference card	17.6%
Young woman specific online forum	15.1%
Poster summarising key facts	11.2%
YouTube videos about healthy eating during pregnancy	6.8%
Telephone or email helpline	6.8%

## Health care professionals questionnaire

### Respondent characteristics

The 146 respondents were mainly midwives (hospital based, community, specialist teenage pregnancy), FNPs (with health visitor, nursing or midwifery background) or health support workers, qualifying between 1972 and 2012 (Table 5).

**Table 5:** Job role of health care professionals responding to the questionnaire (*N* = 146)

	Number	Percent
Any midwife	46	32
Family Nurse Practitioner (FNP)	84	58
Health visitor	2	1
Other professional	1	1
Any support worker	12	8

FNPs were mainly Band 7/8 and midwives were mainly 6/7. Geographical location was not explicitly asked but qualitative answers suggest one respondent works in the Channel Islands (where Healthy Start is not applicable), one near Castleford and others mentioning schemes in London boroughs.

This paper focuses on midwives and FNPs (90% of respondents) as they provide different models of care to young mothers during the antenatal period, and can be related back to findings from the interviews.

## Use of supplements

**Fig. 6:**
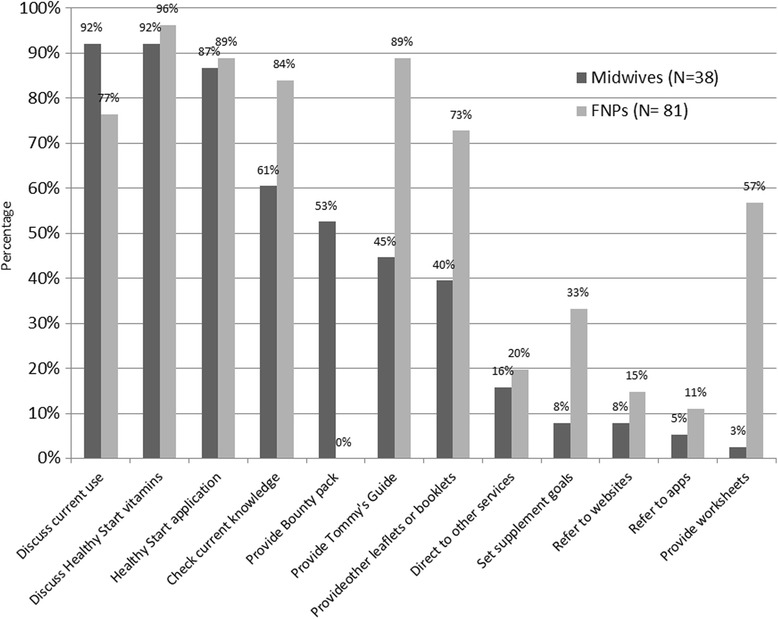
Responses to the question “How do you provide information on supplementation” by job role (*N* = 119)

Nearly all (98.4%) of HCPs provided information about pregnancy supplements for young women, however there were differences in how this information was delivered depending on job role. Midwives were more likely than FNPs to discuss current supplement use and provide a bounty pack whereas FNPs were more likely to provide young women with a copy of the Tommy’s ‘Young Woman's Guide to a Healthy Pregnancy and other leaflets and booklets and were more likely to help with goal setting or provide worksheets. Differences in how professions provided information on supplements are summarised in Fig. 6.

**Fig. 7:**
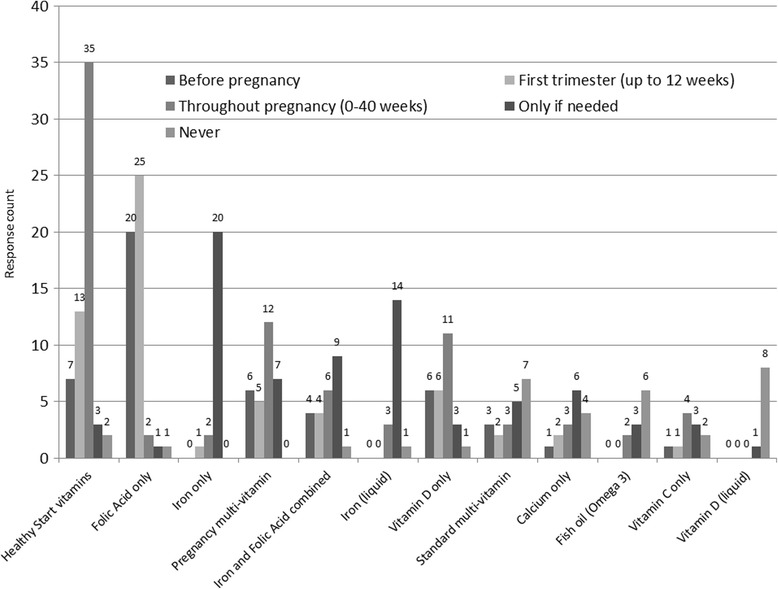
Responses to the question “How often does your documentation remind you to discuss healthy eating and supplements?” by job role (*N* = 115)

When asked if they had any comments on young women accessing Healthy Start vouchers and vitamins both midwives and FNPs reported issues, particularly with application, redemption of vouchers, eligibility criteria being too restrictive, lack of supply in local area and access to collect. In some areas they could hand bottles directly to women and this was seen as a good system. Others noted that it is those clients who are most motivated that take supplements and so they are likely to buy them from a supermarket to have immediately, rather than apply and wait for their vouchers to come through. Discussions regarding supplements and healthy eating were more likely to be prompted by documentation earlier in the pregnancy with midwives, usually at the booking visit (their longest appointment). Young women are not referred to FNPs until later in the pregnancy (approximately 14 weeks), however prompts to discuss diet and nutrition then occurred at most visits as shown in Fig. 7.

## Food choices and dietary advice

Information about healthy eating during pregnancy was provided by 98% of midwives and 100% of FNPs, but how this information was provided differed. The FNPs have more time compared to midwives for goal setting (87% vs 14%) and provided more physical resources such as Tommy’s Young Woman's Guide to Pregnancy (96% vs 57%) providing other leaflets (94% vs 71%) and worksheets (88% vs 1%) which form a major part of the FNP programme. Bounty packs were handed out by 48% of midwives but no FNPs.

**Fig. 8:**
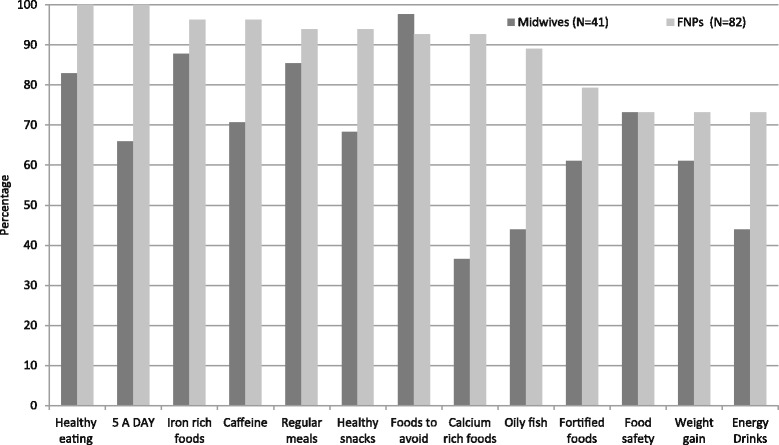
Food topics discussed with young women by health care professionals (*N* = 123)

The food topics discussed with young women by health care professionals are shown in Fig. 8. All 13 food topics were covered by at least 70% of FNPs, but only six (healthy eating, regular meals, iron rich foods, food safety, foods to avoid and caffeine) were covered by at least 70% of midwives. Those less often covered by midwives were energy drinks, healthy snacks, 5 A Day, fortified foods, calcium foods, oily fish and weight gain.

## Confidence and training

Health professionals claimed they felt confident discussing healthy eating and most supplements, however were less confident discussing omega 3, multi vitamins and gestational weight gain. This was particularly true for FNPs and is reflected in the topics that they would like further training on. Overall the demand for additional training was relatively low however the topics which professionals in different roles would like training in differed substantially. There was a higher demand for training among FNPs, particularly on Omega-3 fatty acids and gestational weight gain, whereas midwives most commonly requested training on vitamin D as shown in Table 6.

**Table 6:** Responses to the question “How confident do you feel discussing the following topics with young women?” (*N* = 108)

	Midwives			FNPs		
	Confident	Not confident	Would like training	Confident	Not confident	Would like training
Folic Acid supplements	97%	3%	0%	88%	3%	7%
Good dietary sources of Folic Acid/Folates	89%	9%	3%	87%	8%	4%
Vitamin D supplements	86%	6%	9%	89%	8%	5%
Other sources of Vitamin D	71%	21%	9%	91%	7%	4%
Iron supplements	97%	0%	3%	92%	7%	3%
Good dietary sources of Iron	97%	0%	3%	99%	0%	1%
Healthy Start	94%	6%	0%	97%	0%	3%
Pregnancy multi-vitamins	83%	14%	3%	75%	18%	8%
Omega 3 (fish oils)	56%	35%	9%	62%	25%	15%
General healthy eating guidelines for pregnancy	100%	0%	0%	97%	3%	1%
Foods to avoid during pregnancy	100%	0%	0%	93%	7%	4%
Caffeine	100%	0%	0%	96%	4%	3%
Alcohol	100%	3%	0%	99%	4%	1%
Gestational weight gain	88%	12%	3%	72%	17%	11%

All FNPs and 85% of midwives feel they adequately cover nutrition during pregnancy. The midwives who thought they didn’t adequately cover it stated time pressure (4) and lack of clear guidelines as reasons.

When asked where they accessed information on diet and supplements, they reported accessing dietary advice from most sources listed (90–100%) including dieticians, internal and external training, journals, media and slimming clubs; while less sources were used to access supplement information (17–55%). Only 38% of midwives and 55% of FNPs learnt about supplements in prequalification training, however this may be due to some qualifying decades ago when the recommendations were different.

## Discussion

This cross-sectional study of young mothers and their health care providers gives an insight into the pattern of dietary changes, supplement intake, current sources of information and preferred information sources in addition to the reasons behind their preferences for information support. Addressing eating behaviour and nutritional requirements of adolescent pregnant women in order to enhance their pregnancy and birth experiences is important as an association between poor nutritional status and adverse pregnancy outcomes is well documented [2–4, 15, 16]. This survey allowed a wider verification of the identified issues in a series of in-depth explorative interviews of young women and health care professionals from Doncaster, Manchester and London, details of which are reported elsewhere [11].

These surveys which included responses from all regions in England as well as Scotland, Wales and Northern Ireland showed that young mothers are motivated and keen in adopting healthy dietary changes as indicated in their responses for making positive changes during pregnancy. However it was important that a considerable proportion of responding women were not consuming red meat, eggs, oily fish and soft cheese (such as cream cheese or spreadable cheese). It was encouraging to see a high level of interest and enthusiasm for making positive changes however such a high proportion of adolescents indicating avoiding foods which are potentially valuable sources of vitamins and minerals, such as B vitamins and iron (red meat and eggs) or vitamin D and calcium (oily fish and soft cheese) [17] are of concern. Attention to adequate nutrition in supplying sufficient amount of nutrients are increasingly realised due to its impact on healthy pregnancy and birth outcomes [18].

The poor dietary habit identified in this study is in line with other investigators’ findings showing a poor nutritional intake in young mothers [1, 8]. The low intake of foods such as soft cheese and eggs may suggest inadequate communication with young women and a state of confusion regarding prohibiting raw/runny eggs or unpasteurised mould ripened soft cheeses rather than avoiding all types of eggs or pasteurised soft cheeses. It is likely that food avoidance be due to personal taste or cultural restrictions in which case health professionals’ role in guiding for alternative sources becomes essential for all particularly this group of women.

It is therefore of prime importance to provide sufficient support and appropriate education for health professionals who have direct contact with young pregnant mothers to enhance their knowledge and dietary behaviour with the overall aim of optimising pregnancy and birth outcomes. This is particularly noteworthy, considering the level of awareness and appreciation of the role, and the trust these young women place in their health professionals, especially in midwives, to obtain nutritional information. This ultimately affirms the need for equipping health professionals with appropriate support, skills and knowledge for effective communication of nutritional messages during pregnancy and postpartum.

The majority of midwives and family nurse practitioners felt that they were providing nutritional support to young women during pregnancy as part of their role but acknowledged there were some areas where they lacked confidence and would like further training. The role of midwives in promoting dietary changes has been explored in previous studies and it has been shown to be central in supporting women to make positive changes [19, 20]. The qualitative interviews with young women and health professionals undertaken as part of the earlier stages of this study however found that while most health care professionals provided some nutritional support and information, the extent to which this was tailored to young women's individual needs varied considerably [11]. There are significant barriers to providing individualised nutritional support particularly in terms of time pressures and a lack of appropriate resources [21]. It is therefore important that the need for post-registration training, both relating to the specific nutritional issues identified in this study and wider skills regarding how behaviour change techniques can be applied in this context [22].

There were differences in the resources provided by midwives and FNPs to young women and also in the resources used for their own information. These differences however largely reflect the different models of support and care offered by the different professions. This is further supported by open answer comments from young women, although indicating a high satisfaction and trust in midwifery care, expressing a sense of time limitation or insufficient resources to provide adequate and appropriate support during pregnancy. Young women were aware of risks and biases associated with accessing information via websites or commercial sources due to a lack of consistency and trustworthiness. The findings however were indicative of their desire to receive specific, robust, trustworthy and standardised information from authoritative sources; the potential for alternative digital formats is also indicated via mobile technology (e.g. apps) or interactive websites (e.g. easy to follow recipes, healthy eating guidance). Although they acknowledged mobile technology and digital sources should not replace face to face contacts, they appreciated additional support to allow continuous access, facilitate healthy dietary change and maximise benefits in between appointments. A lack of using online technologies either as tools to facilitate discussion or for helping young women to access further support was evident in the data collected from health care professionals. Young women have been found to routinely access online information [23] and support [24] during pregnancy; however research has suggested that health care professionals lack confidence in using these technologies effectively [25]. This suggests that further investigation to develop and evaluate dietary and lifestyle interventions delivered through mobile technology and interactive websites as an adjunct to care provided by health care professionals may be advantageous.

### Limitations and strengths

The majority of young women respondents were aged over 20 years at the time of the survey; however young women were invited to participate if they aged between 16 and 20 during their pregnancies.

Young women were asked their current age and number of pregnancies, but were not asked to specify their age when they had their first child, so those ‘20 or over’ at the time of the survey, may have had their first child as an adolescent.

Although we asked participants to respond if they were pregnant or recently have been pregnant, the time since the pregnancy may also have varied between participants meaning that some young women may be less likely to remember details about their diet or supplement use accurately. However, the overall dietary habit rather than details of dietary intake was explored which may have less of an impact on the availability of the information. Any pregnancy complications experienced may also have affected the young women's ability to recall information accurately however pregnancy complications were not recorded. With the exception that none of our participants were from Chinese origin, the participants’ ethnic backgrounds were mainly in line with the national data as a great majority have identified themselves as white and a much smaller proportions were from black, mixed and Asian ethnic minorities (national data includes 2.0, 84.4, 2.7, 2.0, 2.1% respectively) [26].

Our study included a self-selected group of young pregnant mothers or those who recently gave birth and this limits the representativeness of our findings; this may have been a more motivated and interested group of young women to respond to such a survey. However, the variation in the included ethnicities in line with the national data regarding ethnicities and postcode data indicating inclusivity of all geographical regions within the UK is encouraging. This as well as the nature of provided information in identifying the poor quality of nutritional behaviour of a considerable proportion of young mothers and their desire for making healthy changes confirms the need for developing appropriate supportive interventions delivered through health care professionals.

Similarly with respect to the health care professionals’ survey it was not possible to calculate a response rate or record the number or characteristics of non-responders to determine if they differed in any way from respondents. This may also have had an impact in that those professionals least comfortable with using online resources may have been less likely to complete the questionnaire. However using online networks allows a remote access with a possibility of open and honest answers without interference or the pressures sometimes felt in the face to face or research situations with close contacts [27].

## Conclusions

A stated positive change in dietary intake by majority of young women in this survey indicates willingness to adapt a healthy lifestyle. This in addition to their trust in health professionals provides an opportunity for health interventions to improve birth outcomes. Avoiding or reducing foods such as red meat, eggs, oily fish or soft cheeses as major sources of required vitamins and minerals is of concern. Improving confidence among health professionals in using a range of resources, including online technologies, to support young women would be advantageous.

### Implications for practice

Providing support for communicating appropriate knowledge and information regarding diet and supplement use to improve nutritional health of this vulnerable group of women is essential. Independent and trustworthy resources for women and professionals, available in a range of formats, providing up to date information which is accessible in between appointments would also be beneficial.

## Abbreviations

FNP: Family nurse practitioner
NDNS: National diet and nutrition survey
NHS: National health service
SD: Standard deviation
UK: United Kingdom
YMCA: The young women at the local young men Christian association
